# Whole Genome Characterization of the Mechanisms of Daptomycin Resistance in Clinical and Laboratory Derived Isolates of *Staphylococcus aureus*

**DOI:** 10.1371/journal.pone.0028316

**Published:** 2012-01-06

**Authors:** Anton Y. Peleg, Spiros Miyakis, Doyle V. Ward, Ashlee M. Earl, Aileen Rubio, David R. Cameron, Satish Pillai, Robert C. Moellering, George M. Eliopoulos

**Affiliations:** 1 Department of Microbiology, School of Biomedical Sciences, Monash University, Melbourne, Australia; 2 Department of Infectious Diseases, The Alfred Hospital, Melbourne, Australia; 3 Division of Infectious Diseases, Beth Israel Deaconess Medical Center, Boston, Massachusetts, United States of America; 4 Harvard Medical School, Boston, Massachusetts, United States of America; 5 3rd Department of Medicine, Aristotle University, Thessaloniki, Greece; 6 Broad Institute, Boston, Massachusetts, United States of America; 7 Cubist Pharmaceuticals, Boston, Massachusetts, United States of America; University of Hyderabad, India

## Abstract

**Background:**

Daptomycin remains one of our last-line anti-staphylococcal agents. This study aims to characterize the genetic evolution to daptomycin resistance in *S. aureus*.

**Methods:**

Whole genome sequencing was performed on a unique collection of isogenic, clinical (21 strains) and laboratory (12 strains) derived strains that had been exposed to daptomycin and developed daptomycin-nonsusceptibility. Electron microscopy (EM) and lipid membrane studies were performed on selected isolates.

**Results:**

On average, six coding region mutations were observed across the genome in the clinical daptomycin exposed strains, whereas only two mutations on average were seen in the laboratory exposed pairs. All daptomycin-nonsusceptible strains had a mutation in a phospholipid biosynthesis gene. This included mutations in the previously described *mprF* gene, but also in other phospholipid biosynthesis genes, including cardiolipin synthase (*cls2*) and CDP-diacylglycerol-glycerol-3-phosphate 3-phosphatidyltransferase (*pgsA*). EM and lipid membrane composition analyses on two clinical pairs showed that the daptomycin-nonsusceptible strains had a thicker cell wall and an increase in membrane lysyl-phosphatidylglycerol.

**Conclusion:**

Point mutations in genes coding for membrane phospholipids are associated with the development of reduced susceptibility to daptomycin in *S. aureus*. Mutations in *cls2* and *pgsA* appear to be new genetic mechanisms affecting daptomycin susceptibility in *S. aureus*.

## Introduction

*Staphylococcus aureus* is one of the most significant human bacterial pathogens, causing immense morbidity and mortality in hospitalised patients, as well as in the community. It causes a diverse range of clinical disease, with mortality from infection reported as high as 35% [1]. Compounding this severity of disease is the constant adaptation of the bacteria to antibiotic pressure, with the emergence of resistance in *S. aureus* now being one of the most important public health problems in the developed world. As a consequence of this resistance, reliance on ‘last-line’ anti-staphylococcal agents such as vancomycin, linezolid, and daptomycin has increased dramatically over recent years, and unfortunately, reduced susceptibility to these agents has also been described [2], [3].

Daptomycin is a cyclic lipopeptide antibiotic that has recently been FDA approved for the treatment of complicated skin and soft tissue infections, and *S. aureus* bacteremia with or without right-sided endocarditis [2]. Understanding the mechanism of action of daptomycin has remained challenging, but current evidence indicates that it interacts with the bacterial cytoplasmic membrane in a calcium-dependent manner, leading to potassium efflux and membrane depolarisation, with subsequent cell death [4], [5]. Using model membranes, it has been shown that negatively charged lipids in the presence of calcium allow daptomycin to insert and perturb bilayer membranes [4]. Importantly, the majority of *S. aureus* membrane lipids are comprised of negatively charged phospholipids: phosphatidylglycerol (PG) and cardiolipin [4].

Therapeutic failures with daptomycin for infections due to *S. aureus* have now been reported, with many of these being associated with the evolution of reduced susceptibility to daptomycin in the setting of deep-seated and poorly controlled infection [2], [6], [7], [8]. The mechanisms of daptomycin resistance in *S. aureus* have focused on the role of the staphylococcal membrane protein, MprF. MprF is a bifunctional protein that mediates both the lysinylation of PG and its translocation to the outer leaflet of the membrane [9]. Point mutations within MprF have been described in *S. aureus* strains with reduced susceptibility to daptomycin, and such mutations appear to cause a gain-in-function, hypothesized to result in accelerated membrane translocation of Lysyl-PG (L-PG), thereby resulting in a reduced net-negative membrane charge that may electrostatically repel calcium-complexed daptomycin [10]. Other mutations that have been reported include mutations in *walK* (previously *yycG*), which encodes a sensor histidine kinase that regulates cell wall metabolism and virulence, and a point mutation in each of *rpoB* and *rpoC*, encoding subunits of RNA polymerase [11]. Furthermore, increased expression of the *dltABCD* operon, which is responsible for D-alanylating wall teichoic acids and contributes to the net-positive surface charge, has been shown to be associated with reduced susceptibility to daptomycin [10]. Thus far, the study of the genetic mechanisms of daptomycin resistance have been limited to analyses of single pairs of isolates or isolates that are laboratory derived [11], [12], [13], [14].

To elucidate the genetic factors involved in the evolution of reduced susceptibility to daptomycin in *S. aureus*, we performed whole genome sequencing of 33 carefully selected strains, which included nine clinical isogenic pairs or series (21 strains) and nine laboratory derived mutants from three parent strains (12 strains). We identified previously described mutations associated with daptomycin resistance such as *mprF*, *walK* and *rpoB* but most importantly, we identified novel mutations in two genes responsible for the production of the anionic membrane phospholipids, PG and cardiolipin. In select clinical pairs, these mutations were associated with changes in phospholipid membrane composition that would explain a reduced affinity for daptomycin, and changes in cell wall thickness. This study represents the first large-scale comparative assessment of genome-wide factors involved in daptomycin-nonsusceptibility in staphylococci derived from active clinical infections.

## Methods

### Ethics Statement

The clinical *S. aureus* isolates used in this study were referred to our laboratory due to persistence during daptomycin therapy. Ethics approval was not required for this laboratory study, as no patient identifiers or clinical details apart from the site where the culture was taken (eg blood, bone or heart valve) was obtained. As referenced in Table 1, some of the strains were from previously published work and some of these publications contain clinical information.

**Table 1:** Characteristics of the daptomycin-exposed clinical strains of *Staphylococcus aureus* used in this study.

Dp-exposed pairs/series	Clinical syndrome	Antibiotic susceptibility		MLST Type	Source
		Dp MIC (ug/ml)	Vn MIC (ug/ml)		
1) A8819	Bacteremia,	0.25	1	105	Boston
A8817	OM, Septic arthritis	2	1		
2) A10102	Bacteremia	0.5	1	5	[2]
A10103		2	1		
3) A9299	Bacteremia	0.25	1–2	5	New York
A9305	Endocarditis	2	1		
4) A9719	Bacteremia,	0.25	1–2	5	Western
A9744	Endocarditis	2	2		Massachusetts
5) A9754	Bacteremia	0.5	2	8	Boston
A9757	Endocarditis	4	2		
6) A8796	Bacteremia	0.5	1	105	Boston [7]
A8799	Vertebral OM	2	2		
7) A9763	Bacteremia	0.5	1	5	Chicago [6]
A9764	OM, PJI	4	2		
8) A9765	Bacteremia	0.5	1	8	Chicago [6]
A9766	OM	2	2		
9) A9781	Bacteremia	0.5	1	5	Boston
A9784		0.5			
A9788		1			
A9792		2			
A9798		2	2		

Dp, daptomycin; MLST, multi-locus sequence type; OM, osteomyelitis; PJI, prosthetic joint infection; Vn, vancomycin.

### Bacterial strains and culture conditions

Isolates obtained from the same patient were previously confirmed to be isogenic based on pulsed-field gel electrophoresis. All bacteria were stored at −80°C until further testing. Daptomycin (Cubist, MA) susceptibility testing was performed by broth macrodilution using cation-adjusted Mueller-Hinton II broth (BD) supplemented to contain a final calcium concentration of 50 µg/ml. To determine the differences between *in vivo* and *in vitro* daptomycin exposure on the genetic evolution to resistance, three reference *S. aureus* strains underwent *in vitro* daptomycin exposure. A prototype hospital-acquired MRSA strain was selected (MRSA32 [A5948]), as well as an *agr*+ (RN6607 [A8115]) and its isogenic *agr*− mutant strain (RN9120 [A8117]). The latter two strains also enabled an assessment of the effect of *agr* on daptomycin resistance. *In vitro* daptomycin exposure was performed in Brain Heart Infusion (BHI) broth using a high bacterial inoculum (10^8^ CFU/ml) and 48 hour exposure to daptomycin at a concentration of 8 µg/ml at 35°C. The culture was then plated onto agar containing varying concentrations of daptomycin, and single colonies were chosen for formal susceptibility testing. Three independent mutants from each reference strain were selected for sequencing. One of the mutants from A5948 was generated previously (A6658) [15].

### Whole genome sequencing

Genomic DNA was extracted according to manufacturer's guidelines (Promega Wizard Genomic Kit). All daptomycin-susceptible parent genomes were sequenced using 454 FLX pyrosequencing (Roche) with DNA fragment libraries according to the manufacturer's recommendations. Genomes were assembled using Newbler and the runAssembly script was then used to assemble reads into contigs. To remove contaminating sequences, final assemblies were BLASTed to the NCBI non-redundant (NR) database and UniVecCore. Assembly annotation was performed using a combination of *ab initio* and evidence-based approaches. For further details see supplementary material (Text S1). A summary of gene finding data for each locus can be viewed at the Broad Institute *S. aureus* Drug Resistance Project group database (http://www.broadinstitute.org/annotation/genome/staphylococcus_aureus_drug_resistance/).

### Single Nucleotide Polymorphism (SNP) and Phylogenetic Analysis

The daptomycin-nonsusceptible daughter strains were sequenced to high coverage (≥100 fold) with 76 nucleotide reads produced on the Illumina platform. The reads were used to call SNPs against the parent assemblies using the variant ascertainment algorithm (VAAL), a polymorphism discovery algorithm for short reads developed by the Broad Institute [16]. Select SNPs were confirmed independently using PCR sequencing. Phylogenetic analysis was performed using single-copy core gene trees that were generated from orthologous groups computed by orthoMCL (http://www.orthomcl.org/cgi-bin/OrthoMclWeb.cgi) [17]. An all versus all BLAST was performed using the predicted protein sequence from all genomes of interest. All BLAST hits with an e-value<1e^−5^ were used as input to orthoMCL. To generate trees, nucleotide sequences for all single-copy core genes were retrieved and aligned by orthologous group [18]. These alignments were trimmed using trimAL to allow concatenation [19]. Aligned, trimmed and concatenated sequences were then used to build trees using FastTree [20]. Using the genome sequence for all parent strains, multi-locus sequence types (MLST) were also determined (http://saureus.mlst.net/). This is a well established method for determining clonality in *S. aureus* and utilizes the sequence of seven house-keeping genes to determine allelic profiles [21]. Predicted transmembrane domains (TMDs) of proteins were determined using TMHMM v 2.0 [22].

### Membrane lipid analysis

Lipid measurements were performed after a modified Bligh and Dyer extraction of the total polar lipids [23] and quantified using LC-MRM (multiple reaction monitoring) as described in supplementary material (Text S1). Three independent colonies from each strain were assessed and each extract was run in triplicate. Differences in the PG∶L-PG ratio were assessed by student's *t* test at a significance level of *P*≤0.05.

### TEM

Cells were prepared for TEM as described previously [5]. In brief, cells from late-exponential phase were fixed with 2.5% (vol/vol) glutaraldehyde and 2.0% (wt/vol) osmium tetroxide, and then stained with 2.0% (wt/vol) uranyl acetate. After embedding in LR White resin and cutting thin sections, samples were further stained with uranyl acetate and then imaged using a LEO 912AB microscope. The cell wall of 100 cells for each strain were measured in a blinded fashion and compared using the student's *t* test with a significance level of *P*≤0.05.

## Results and Discussion

### Clinical and laboratory *S. aureus* strains exposed to daptomycin

The characteristics of the *S. aureus* strains used in this study are shown in Tables 1 and 2. All the clinical pairs or series of *S. aureus* were obtained from patients with bloodstream infection who failed or had persistent infection while being treated with daptomycin. Most were complicated by endocarditis or other deep-seated infection (Table 1). Each pair or series includes the initial infecting isolate, which was daptomycin-susceptible (MIC≤1 µg/ml), and the subsequent isolate/s that were daptomycin-nonsusceptible after daptomycin exposure. For most strains, exposure to daptomycin did not change the MIC to vancomycin (Table 1 and 2). The increase in MIC to daptomycin for the laboratory exposed strains was equivalent to that seen for the clinical isolates, with no differences observed between *agr*+ *S. aureus* (A8115) and its isogenic *agr*− (A8117) mutant strain (Table 2). *In vitro* selection has the advantage of assessing genetic changes specific to daptomycin exposure alone, but is limited by the absence of host factors and immune responses that may alter the evolution of daptomycin resistance. This unique collection of carefully selected isogenic *S. aureus* strains provided us with an excellent opportunity to investigate the mechanisms of resistance to one of our last line anti-staphylococcal antibiotics, daptomycin.

**Table 2:** Characteristics of the daptomycin-exposed laboratory strains of *Staphylococcus aureus* used in this study.

Laboratory Dp-exposed pairs^a^	Antibiotic susceptibility		MLST Type	Source
	Dp MIC (ug/ml)	Vn MIC (ug/ml)		
1) A8115^b^	0.5	1	5	R. Novick
A10135	2	1		
A10151	4	1		
A10152	2	1		
2) A8117^c^	0.5	1	5	R. Novick
A10136	2	1		
A10153	2	2		
A10154	2	2		
3) A5948^d^	1	1	8	[15]
A6658	2	1		
A10155	2	1		
A10156	2	0.5		

Dp, daptomycin; MLST, multi-locus sequence type; Vn, vancomycin.

a Three individual mutants were generated from each daptomycin-susceptible laboratory parent strain. The three mutants are represented for each susceptible strain.

b RN6607 (*agr*+).

c RN9120 (*agr*−).

d MRSA32.

### Whole genome sequencing of daptomycin-susceptible *S. aureus* parent strains

All daptomycin-susceptible parent genomes were sequenced to an average of 24-fold coverage (range 14- to 39-fold) and were of similar sizes, ranging from 2.72 Mb to 2.97 Mb (mean of 2.84 Mb). The mean number of putative open reading frames was 2757 (range 2539–3066) and the percentage GC content was similar between strains (mean 32.72%, range 32.58%–32.84%) (http://www.broadinstitute.org/annotation/genome/staphylococcus_aureus_drug_resistance/). As shown in Tables 1 and 2, seven of the parent strains were sequence type (ST) 5 and three were ST8, which are the more common MLST types in hospital-acquired *S. aureus* in North America [24]. Two parent strains were ST105, which has been less commonly reported (http://saureus.mlst.net/). Detailed phylogenetic analysis is shown in Fig. S1, and illustrates the high degree of genetic conservation between the parent strains isolated from different patients in this study.

### Number and type of genetic mutations associated with daptomycin exposure

**Figure 1:**
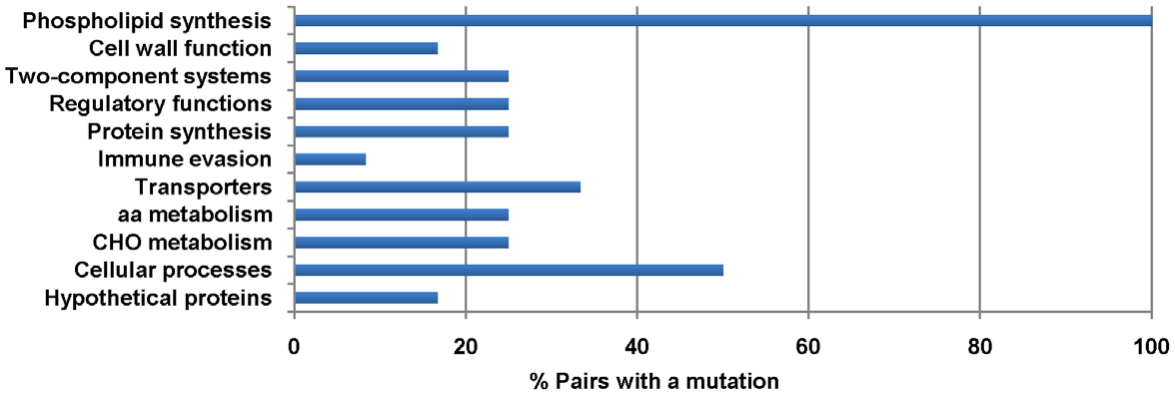
Percentage of daptomycin-exposed pairs (n = 12) with a mutation in each functional gene category. All daptomycin-nonsusceptible isolates had at least one mutation in a gene coding for phospholipid biosynthesis, including *mprF*, *cls2* or *pgsA*.

To investigate the genetic mutations associated with the *in vivo* or *in vitro* evolution of daptomycin resistance, all isogenic daughter strains (21 strains) were sequenced and compared to their daptomycin-susceptible parent strains. On average, only six coding region mutations were observed across the entire genome in the clinical *in vivo* daptomycin exposed strains (range 2–13 mutations), whereas only two mutations on average (range 1–4 mutations) were seen in the laboratory *in vitro* exposed pairs (Table S1). In the clinical pairs, the majority of mutations were SNPs (mean 4, range 2–8), with an average of 2 insertions or deletions per pair. All but three SNPs led to an amino acid change in their respective proteins (Table S1). Mutated genes were categorized by function to identify themes of bacterial physiology that may contribute towards reduced susceptibility to daptomycin (Fig. 1). Interestingly, the most consistent mutation, which was found in all 12 strain pairs, was a mutation in a gene important for membrane phospholipid biosynthesis, highlighting the central aspect of cell membrane physiology to susceptibility to daptomycin.

### Mutations within genes associated with phospholipid biosynthesis

Daptomycin is an anionic molecule but when complexed with calcium ions, its properties and mode of action are analogous to cationic antimicrobial peptides [9], [25], where interaction with the negatively-charged cell membrane leads to membrane perturbation and cell lysis [4], [5]. The gene most frequently mutated in our daptomycin-resistant strains was *mprF*. Point mutations in this gene were found in all nine clinical pairs and one laboratory-derived pair (Table 3 and S1). This gene codes for a large membrane protein that contains 14 transmembrane domains (TMDs), and serves two key functions; the addition of positively charged lysine residues onto PG to form L-PG, which is mediated by a *C*-terminal lysinylation domain, and the translocation of this L-PG to the outer leaflet of the cytoplasmic membrane, which is mediated by the *N*-terminal translocation domain [9]. This acts to neutralize membrane charge and creates resistance to cationic peptides such as host immune factors and daptomycin, and highlights its importance in bacterial immune evasion and fitness within the host [9], [26]. It has been previously shown that *mprF* point mutations in daptomycin-nonsusceptible isolates are associated with a gain-in-function, leading to greater L-PG in the outer leaflet of the membrane and a further reduction in the net-negative membrane charge leading to electrorepulsion [26].

**Table 3:** Predicted protein changes in clinical- and laboratory-derived daptomycin-nonsusceptible isolates of *Staphylococcus aureus*.

Dp-nonsusceptible strains^a^	Predicted Amino acid Change		
	MprF	Cls2	PgsA
**Clinically-derived**			
1) A8817	T345I	F60S	
2) A10103	S295L		
3) A9305	S295L		
4) A9744	S337L	A23V	
5) A9757	I420N		
6) A8799	S337L		
7) A9764	L826F	L52F	
8) A9766	S295L		
9) A9792	S295L		
A9798	G61V		
**Laboratory-derived** b			
1) A10135			A64V
A10151		T33N	
A10152			A64V
2) A10136			S177F
A10153			A64V
A10154			K65R, insert G76, E77
3) A6658	L826F		
A10155		T33N	
A10156			V59N

Dp, daptomycin.

a Each daptomycin-nonsusceptible strain was derived from a daptomycin-susceptible parent strain shown in table 1. The predicted amino acid change relates to the gene mutation between the listed strain and its susceptible parent.

b Three individual mutants were generated from each daptomycin-susceptible laboratory parent strain. The three mutants are represented for each susceptible strain.

**Figure 2:**
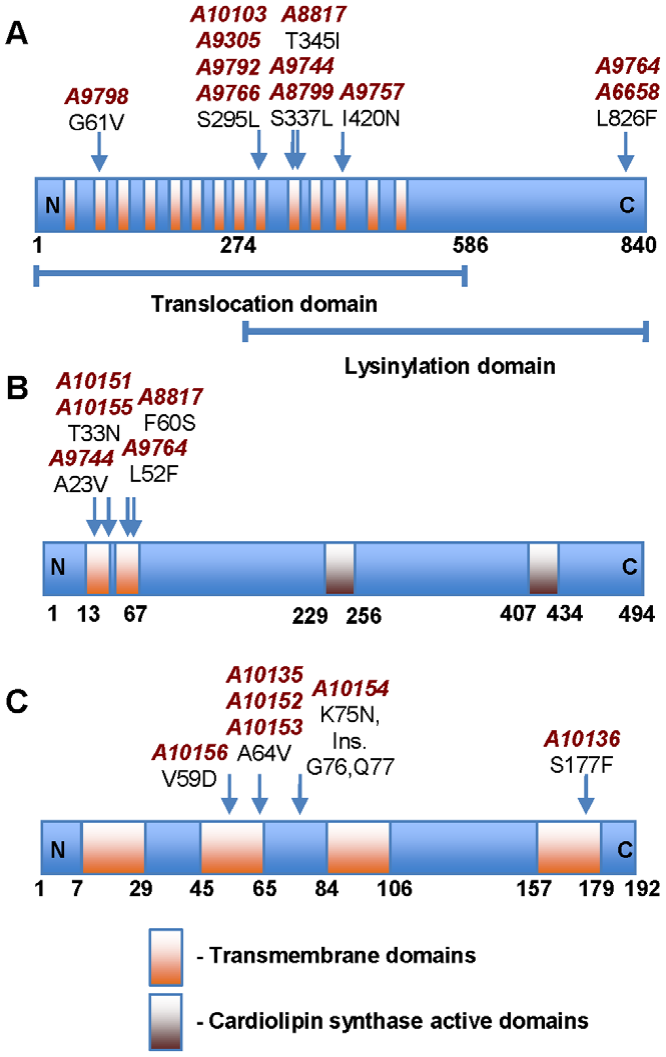
Phospholipid biosynthesis genes are integral to the development of reduced susceptibility to daptomycin in *S. aureus*. (**A**) The predicted amino acid changes associated with the 11 SNPs identified within *mprF*. (**B**) The five mutations identified in *cls2* were mapped to four positions in the protein, all within the two *N*-terminal transmembrane domains. (**C**) Six mutations were identified in *pgsA* and were mapped to four positions in the protein, three of which were in transmembrane domains. The ‘A’ numbers correspond to the daptomycin-nonsusceptible isolates, and the arrows point to the position of the amino acid change.

The predicted amino acid changes associated with the 11 SNPs identified within *mprF* in this study are shown in Fig. 2A. All SNPs were independently confirmed by PCR and sequencing. Eight SNPs were mapped to four positions within the lysinylation and translocation domains (Fig. 2A). One SNP (G61V) was found within the *N*-terminal translocation domain only, and two SNPs were mapped to one position (L826F) in the *C*-terminal lysinylation domain only (Fig. 2A). We hypothesize that the latter two predicted amino acid changes may either directly enhance translocation of L-PG to the bacterial surface by increasing translocase activity of MprF, or enhance lysinylation of PGs leading to increasing pools of L-PG on the inner leaflet of the membrane [9]. Accumulation of intracellular L-PG may stimulate translocation to the outer leaflet of the membrane in a gradient-dependent manner. This would, in turn, exacerbate the reduced net-negative charge of the bacterial surface, thus contributing to daptomycin non-susceptibility [9], [10], [26]. The importance of mutations within MprF to daptomycin susceptibility is also shown by the sequence of mutations in the clinical series (A9781–A9798). As shown in Table 1 and S1, the MIC to daptomycin increased to the nonsusceptible range once the *mprF* mutation occurred in A9792 (S295L). Interestingly, the final isolate of the series (A9798) had a different *mprF* mutation (G61V) compared to its predecessor (A9792), and both strains had mutations in genes not found in the other. This suggests that multiple resistant subpopulations are likely to have been present rather than a step-wise accumulation in mutations. Finally, not all daptomycin-nonsusceptible mutants had *mprF* mutations, particularly the laboratory-derived strains, suggesting that host pressures, such as cationic antimicrobial peptides, may influence MprF-mediated daptomycin resistance.

Mutations in a second phospholipid biosynthesis gene were identified in five of the studied pairs (Tables 3 and S1). This gene was cardiolipin synthase (*cls2*), mutations of which may have been predicted to have a role given observations of altered gene expression in a laboratory-derived daptomycin-nonsusceptible strain [27]. Cardiolipin is an important anionic membrane phospholipid that is synthesized from the phosphatidyl moiety of two PG molecules by the membrane-bound enzyme, Cls [28]. Under conditions of stress, such as unfavorable growth conditions or cell-wall acting antibiotics, cardiolipin can accumulate up to ∼25%–30% of membrane phospholipid [28]. Cardiolipin synthase is predicted to contain two TMDs, spanning residues 13–35 and 45–67, as well as two putative cardiolipin synthase domains across residues 229–256 and 407–434 (Fig. 2B). The four SNPs identified within Cls2 reside exclusively within the two putative TMDs at the *N*-terminus of the protein (Fig. 2B). All mutations were independently confirmed by PCR and sequencing. Importantly, two of the laboratory-derived daptomycin-nonsusceptible mutants (A10151 and A10155) from two distinct parent strains (A8115 and A5948; Tables 3 and S1), had only a single point mutation in *cls2*, both at the same position and involving the same amino acid change (Thr33Asn). This single point mutation in *cls2* caused an increase in MIC to daptomycin from 0.5 µg/ml to ≥2.0 µg/ml in both pairs. No other gene mutations were identified in these two pairs based on the whole genome sequence. We hypothesise that mutations within the TMDs impair membrane localisation and function of Cls, resulting in altered cardiolipin synthesis. These changes alone, or in concert with an *mprF* mutation, which was seen in three of the clinical pairs (Table 3), may be important in the charge-based repulsion of daptomycin, or may alter binding of daptomycin to the membrane. Interestingly, it has previously been shown that *cls* gene expression, as determined by microarray analysis, was repressed in a laboratory-derived daptomycin-nonsusceptible mutant but mutations in the gene were not assessed [27]. Finally, adding strength to the role of *cls* in daptomycin susceptibility, two recent reports have described its involvement in reduced susceptibility to daptomycin in clinical strains of *Enterococcus* [29], [30].

**Figure 3:**
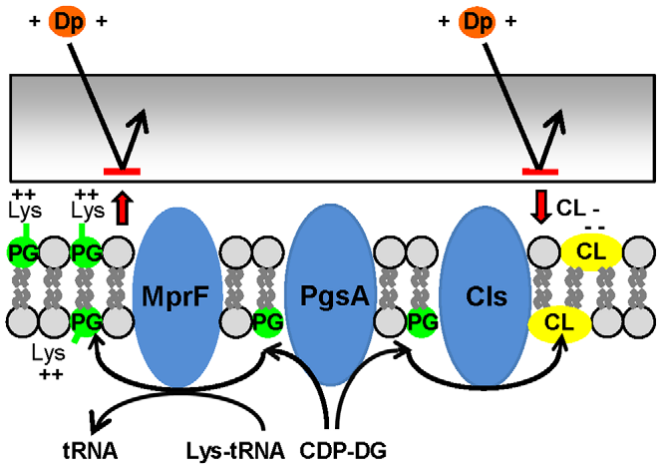
Schematic of our working hypothesis for the functional effect of the observed mutations. *mprF* mutations lead to an increase in lysinylation of phosphotidylglycerol (PG) to form L-PG, and an increase in translocation of this positively charged L-PG to the outer leaflet of the membrane, leading to electrorepulsion of daptomycin. In isolation, or in concert with *mprF* mutations, mutations in *cls2* may lead to altered membrane charge or effect binding of daptomycin to the membrane. Finally, PgsA is important in the initial step of phospholipid biosynthesis, converting CDP-diacylglycerol (CDP-DG) into PG.

A third gene novel to daptomycin susceptibility and also involved in membrane phospholipid biosynthesis was also identified, known as CDP-diacylglycerol-glycerol-3-phosphate 3-phosphatidyltransferase (*pgsA*). PgsA is important in the production of PG, the most abundant anionic membrane phospholipid. Interestingly, mutations in *pgsA* have recently been described to be associated with reduced susceptibility to daptomycin in *Bacillus subtilis* [31]. Our mutations were only identified in laboratory-exposed strains; however as shown for *cls*, a point mutation in *pgsA* alone (strain A10152) was enough to cause an increase in MIC of daptomycin from 0.5 µg/ml to 2 µg/ml (Tables 2 and S1). Five SNPs were mapped to three positions of the protein, and as seen with *cls* mutations, all these SNPs were within TMDs (Fig. 2C). One in-frame insertion was also identified (Table 3 and Fig. 2C). Our hypothesis is that these mutations impair PgsA function leading to reduced PG in the membrane and subsequent surface charge alterations. Further work is required to characterize the role of this gene in *S. aureus* susceptibility to daptomycin. The interaction and significance of mutations in these three phospholipid biosynthesis genes to daptomycin susceptibility is shown in figure 3.

### Host pressure and daptomycin exposure lead to mutations across a functionally diverse array of genes

We observed a greater number of mutations in our clinical versus laboratory-derived pairs, highlighting the importance of host immune pressures on the genetic response of *S. aureus* to an antimicrobial. Apart from mutations in phospholipid biosynthesis genes, five of the clinical pairs had at least one mutation in a gene encoding a transcriptional regulator or a two-component signal transduction system (Fig. 1 and Table S1). These included *walK* (previously *yycG*), *agr*, *stp1*, *tcaR* and *rsbU* (sigma-B regulation), which are all important cell wall biosynthesis and/or virulence regulators, with the majority being reported for the first time relating to daptomycin exposure. Despite mutations in *rpoB* being initially reported with a laboratory-derived daptomycin-nonsusceptible isolate [11], we identified only two pairs (clinical) that had mutations within *rpoB* (Table S1). Less common mutations were observed in other systems of physiological function including carbohydrate and amino acid metabolism, ion and small molecule transporters, and housekeeping genes (Fig. 1 and Table S1), demonstrating the diverse array of genes mutated during the *in vivo* evolution of daptomycin resistance. However, apart from the genes involved in phospholipid biosynthesis, none of the other genetic mutations were consistently observed across all the pairs. It is of interest though that several of the mutated genes regulate cell wall biosynthesis, and have also been implicated in reduced susceptibility to vancomycin [3]. Furthermore, many are associated with virulence regulation, and suggest that these daptomycin-nonsuscpetible clinical isolates may have altered virulence; a hypothesis that needs further evaluation.

### Daptomycin induces cell wall thickening and changes in membrane lipid composition

**Figure 4:**
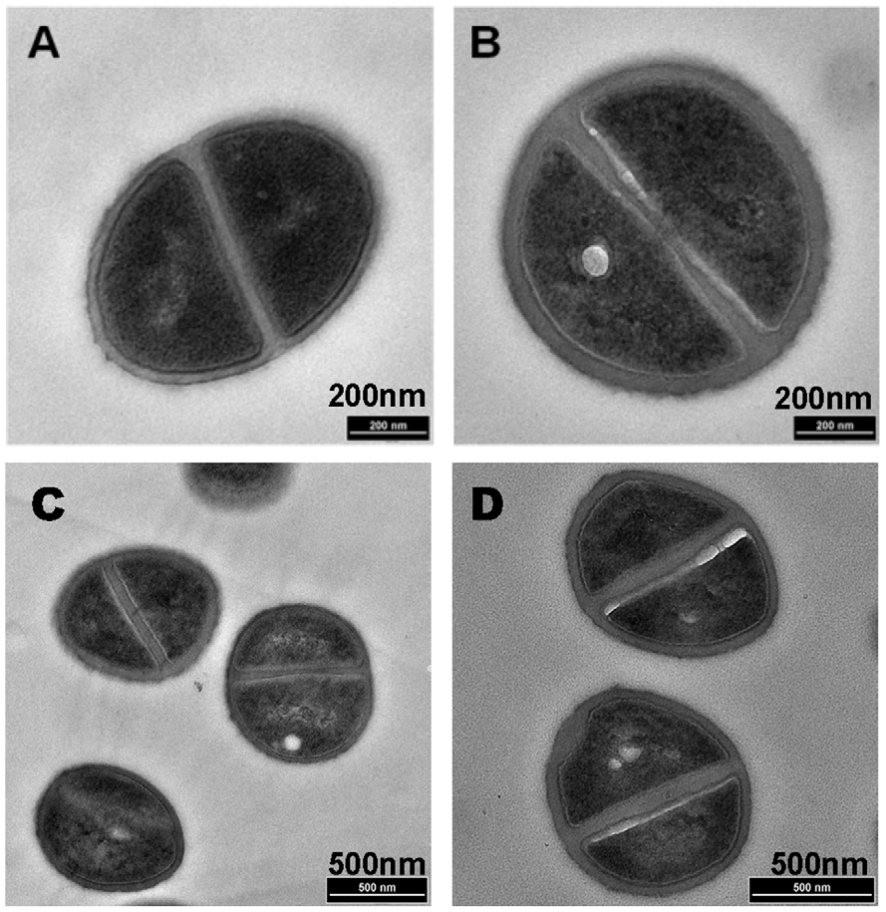
Transmission electron microscopy of two clinical pairs showing a thickening of the cell wall in the daptomycin-nonsusceptible isolates. (A) and (B) represent A8819 (daptomycin-susceptible) and A8817 (daptomycin-nonsusceptible), respectively. (C) and (D) represent A8796 (daptomycin-susceptible) and A8799 (daptomycin-nonsusceptible), respectively. *P*<0.001 for both.

Given the frequency of mutations associated with phospholipid biosynthesis, and the presence of mutations in genes that regulate cell wall turnover (*walK*, *agr*, *stp1*), we characterized the ratio of cell membrane lipids (PG∶L-PG ratio) and cell wall thickness in two representative clinical pairs (A8819/A8817 and A8796/A8799). Both these pairs have point mutations in *mprF* in different locations of the lysinylation domain (Fig. 2A). Consistent with the described physiology, the daptomycin-nonsusceptible daughter strain, A8817, had an increase in L-PG in the cell membrane, with a drop in the PG∶L-PG ratio from 3.7 to 1.2 for A8819/A8817 (*P*<0.05). For the second pair, despite there being an increase in L-PG in absolute terms (PG∶L-PG ratio of 3.2 to 2.5 for A8796/A8799), the difference was not significant (*P* = 0.2). It has previously been shown that subtle changes in L-PG in the outer membrane leaflet can affect daptomycin susceptibility [9]. In the same two clinical pairs, we also observed greater cell wall thickness in both daptomycin-nonsusceptible isolates versus their susceptible parent strains (Fig. 4), with a mean (±SD) thickness of 35.7 nm (±4.1) and 42.8 nm (±5.6) in the susceptible strains (A8819 and A8796, respectively) versus 48.1 nm (±5.9) and 53.2 nm (±7.1) in their corresponding resistant daughter strains (A8817 and A8799, respectively) (*P*<0.001 for both). Of note, the genetic mutations in both these pairs (Table S1) did not clearly explain the increase in cell wall thickness. Other groups have shown that cell wall thickness in daptomycin-nonsusceptible strains is not a consistent finding [14], [32], and a more recent analysis suggested that in some strains it may be due to an increase in wall teichoic acid [33].

### Conclusions

We have performed whole genome sequencing of the largest collection of daptomycin exposed *S. aureus* strains to date. Our data show that mutations in genes responsible for phospholipid biosynthesis appear important for the development of reduced susceptibility to daptomycin, more specifically, mutations in *mprF*, *cls2* and *pgsA*. We hypothesise that mutation in each of these genes act similarly to reduce the net-negative charge of the cell membrane leading to electrorepulsion of daptomycin. They may act in isolation or in concert with each other, particularly for mutations in *mprF* and *cls2* (Fig. 3). Our data also show that *in vivo* pressures in the setting of daptomycin exposure select for a range of other genetic mutations, including those involving virulence regulatory genes. Finally, we have shown in select clinical isolates, the functional significance of the observed genetic mutations by analysing changes in the cell wall and membrane lipid profiles. Through use of carefully selected strains and broad-based genomics, this work provides important insights into the mechanism of resistance to one of our last-line anti-staphylococcal antibiotics, daptomycin.

## Supporting Information

Figure S1 **Phylogenetic analysis of 1230 common single copy genes found in 12 daptomycin-susceptible** ***Staphylococcus aureus*** **parent strains, with** ***Staphylococcus epidermidis*** **ATCC 12228 and RP62A used as outgroups for the analysis.**
(PPT)

Table S1 **Mutations identified between daptomycin-susceptible and isogenic daptomycin-nonsusceptible strains of** ***Staphylococcus aureus*** **.**
(DOC)

Text S1 **More detailed methods.**
(DOCX)
