# Supplementary figures and images for: Whole Genome Characterization of the Mechanisms of Daptomycin Resistance in Clinical and Laboratory Derived Isolates of Staphylococcus aureus
Source: PLoS One. 2012 Jan 6;7(1):e28316. doi: 10.1371/journal.pone.0028316 (PMC3253072; doi:10.1371/journal.pone.0028316)

## Slide 1
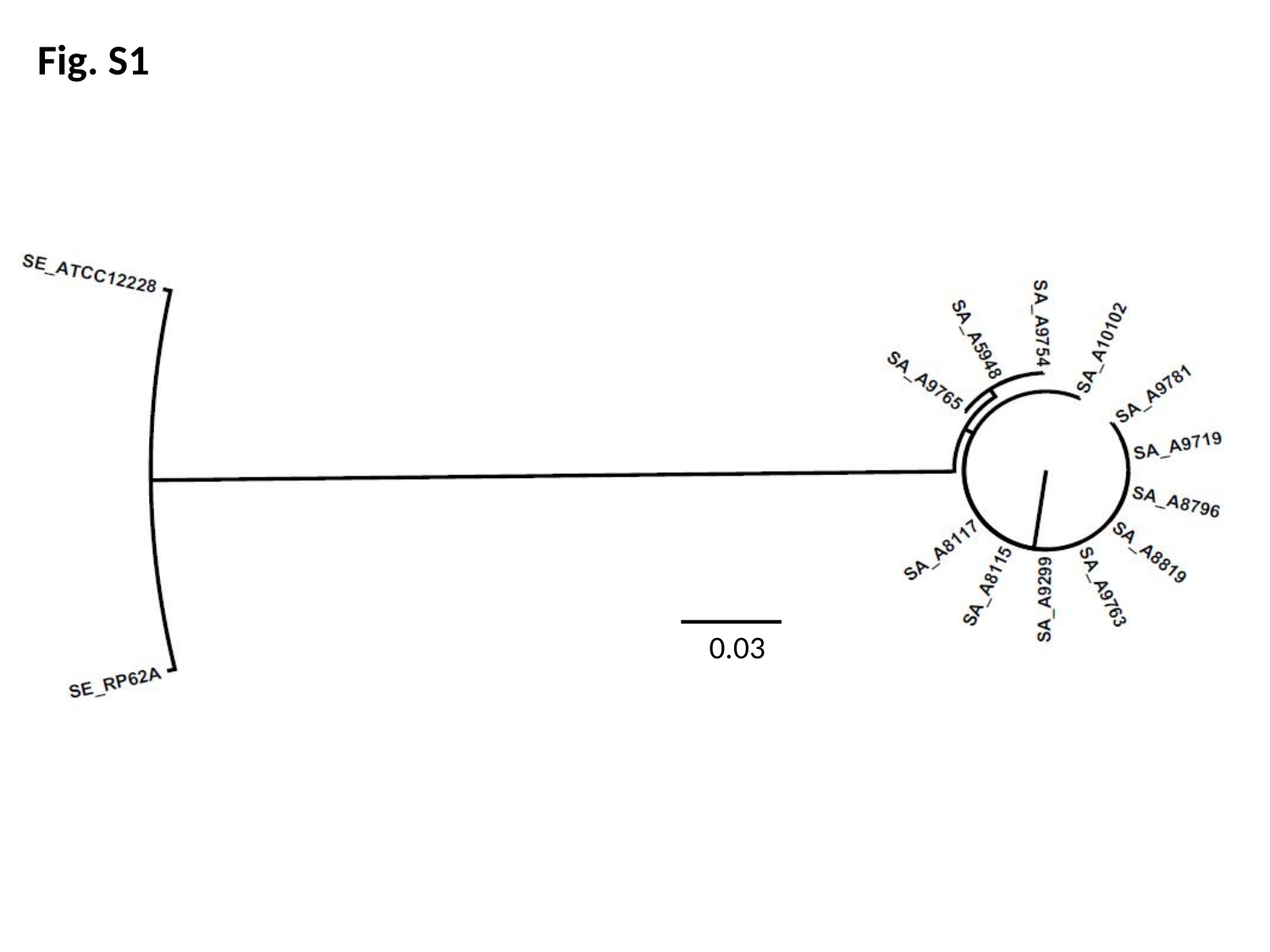

Fig. S1
0.03

Supplement: Figure S1 — Phylogenetic analysis of 1230 common single copy genes found in 12 daptomycin-susceptible Staphylococcus aureus parent strains, with Staphylococcus epidermidis ATCC 12228 and RP62A used as outgroups for the analysis. (PPT) [file pone.0028316.s001.ppt]
