# Supplementary material for: Whole Genome Characterization of the Mechanisms of Daptomycin Resistance in Clinical and Laboratory Derived Isolates of Staphylococcus aureus
Source: PLoS One. 2012 Jan 6;7(1):e28316. doi: 10.1371/journal.pone.0028316 (PMC3253072; doi:10.1371/journal.pone.0028316)
Supplement: Table S1 — Mutations identified between daptomycin-susceptible and isogenic daptomycin-nonsusceptible strains of Staphylococcus aureus . (DOC) [file pone.0028316.s002.doc]

**SUPPLEMENTARY MATERIAL – Table S1**

**Table S1** – Mutations identified between daptomycin-susceptible and isogenic daptomycin-nonsusceptible strains of *S. aureus*.

| **Daptomycin-exposed pairs** | | **Family** | **Gene*^a^*** | **Protein** | **Known/putative function** | **Size*^b^*** | | **Mutations*^c^*** | | |
| --- | --- | --- | --- | --- | --- | --- | --- | --- | --- | --- |
| **S** | **NS** |  |  |  |  | **bp** | **aa** | **Type of mutation** | **Nucleotide (s)** | **Protein** |
| **Clinical Pairs** | |  |  |  |  |  |  |  |  |  |
| **A8819** | **A8817** | Phospholipid synthesis | *mprF* | Lysylphosphatidylglycerol synthetase | Oxacillin resistance related protein | 2523 | 840 | SNP | 1034, C 🡪 T | Thr345Ile |
|  |  |  | *cls2* | Cardiolipin synthetase | Phosphatidylcholine-hydrolysing phospholipase | 1485 | 494 | SNP | 179, T 🡪 C | Phe60Ser |
|  |  | Amino acid metabolism | *leuS* | Leucyl-tRNA synthetase | Incorperation of leucine into tRNA | 2418 | 805 | SNP | 1402, A 🡪 G | Asn468Asp |
|  |  | Carbohydrate metabolism | *mnaA* | UDP-N-acetylglucosamine 2-epimerase | Sialic acid synthesis | 1131 | 376 | SNP | 906, A 🡪 C | Arg302Ser |
|  |  | Cellular processes | *ispA* | Geranyltranstransferase | Polyketide biosynthesis | 882 | 293 | Deletion | 422-841 | Mis-sense frameshift from Leu141His, truncates protein to 145aa |
|  |  |  |  |  |  |  |  |  |  |  |
| **A10102** | **A10103** | Phospholipid synthesis | *mprF* | Lysylphosphatidylglycerol synthetase | Oxacillin resistance related protein | 2523 | 840 | SNP | 884, C 🡪 T | Ser295Leu |
|  |  | Regulatory functions | *rsbU* | Sigma-B regulation protein RsbU | Phosphoserine phosphatase, activates *sigB* regulon | 1002 | 333 | Insertion | 887, ACGTC | Mis-sense frameshift from Asp39Gly, truncates protein to 41aa |
|  |  | Immune evasion | *scn* | Staphylococcal complement inhibitor | Complement inhibitor C3 convertase | 358 | 97 | Deletion | 65-71, CTAATCT | Gain of function frameshift, Stop to Asp25, extends protein to 116aa |
|  |  | Transporters | *lctP* | Lactate transporter | L-lactate permease/lactate transport | 1602 | 533 | Deletion | 1322-1324, TGT | In-frame deletion from Leu442Phe |
|  |  |  | *feoB* | Ferrous iron transporter B | Integral membrane protein required for iron transport | 1995 | 664 | SNP | 1906, A 🡪 G | Lys636Glu |
|  |  |  | *opuD* | Glycine betaine transporter | BCCT family transporter | 1648 | 504 | Deletion | 1465, C | Mis-sense frameshift from Val489, extends protein to 528aa |
|  |  |  | SA0138 | Phosphonate ABC transporter | Phosphate/phosphonate transport system substrate-binding protein | 926 | 218 | Deletion | 325-329, ACGGC | Mis-sense frameshift from Arg111Tyr, extends protein to 318aa |
|  |  | Amino acid metabolism | *gltB* | Glutamate synthase/ferredoxin | Glutamate synthesis, glutamate synthase subunit | 4500 | 1499 | Insertion | 779, GCACGCC | Mis-sense frameshift from Gln260Arg, truncates protein to 263aa |
|  |  |  | *aroH/aroA* | Bifunctional 3-deoxy-7-phosphoheptulonate synthase/chorismate mutase | Aromatic amino acid synthesis | 1092 | 363 | SNP | 902, A 🡪 G | Lys301Arg |
|  |  | Cellular processes | *rimL* | Acetyltransferase, RimL | Transfers acetyl group from acetyl-CoA to primary amine of substrates | 543 | 180 | Insertion | 520, GC | Mis-sense frameshift from Tyr174Ala, extends protein to 192aa |
|  |  |  | *pdxS* | Pyridoxine biosynthesis protein | Vitamin B6 synthesis /subunit of the pyridoxal 5'-phosphate synthase, SOR/SNZ family | 894 | 297 | Deletion | 299-304, GTGTTG | In-frame deletion of Gly100 and Val101 to Asp100 |
|  |  |  | SA0754 | Acetyltransferase | Transfers acetyl group from acetyl-CoA to primary amine of substrates | 495 | 164 | SNP | 415, A 🡪 G | Ser139Gly |
|  |  |  | *pre* | Plasmid recombination enzyme type 2 | Conjugative mobilisation | 1263 | 420 | Insertion | 1065, AC | Mis-sense frameshift from Lys356Leu, truncates protein to 366aa |
|  |  |  | SAV0879 | Conserved hypothetical protein | Homologous to Staphylococcal phage proteins of unknown function | 390 | 129 | SNP | 171, C 🡪 T | Asn57Asn |
|  |  |  |  |  |  |  |  |  |  |  |
| **A9299** | **A9305** | Phospholipid synthesis | *mprF* | Lysylphosphatidylglycerol synthetase | Oxacillin resistance related protein | 2523 | 840 | SNP | 884, C 🡪 T | Ser295Leu |
|  |  | Protein synthesis | *era* | *E. coli* Ras-like protein | GTP-binding protein, binds 16S rRNA and 30S ribosome | 900 | 299 | SNP | 553, C 🡪 T | His185Tyr |
|  |  |  |  |  |  |  |  |  |  |  |
| **A9719** | **A9744** | Phospholipid synthesis | *mprF* | Lysylphosphatidylglycerol synthetase | Oxacillin resistance related protein | 2523 | 840 | SNP | 1010, C 🡪 T | Ser337Leu |
|  |  |  | *cls2* | Cardiolipin synthetase | Phosphatidylcholine-hydrolysing phospholipase D | 1485 | 494 | SNP | 68, C 🡪 T | Ala23Val |
|  |  | Cell wall function | *atl* | N-acetylmuramoyl-L-alanine amidase | Bi-funtional autolysin involved in cell envelope biogenesis | 3768 | 1255 | Insertion | 1430, A | Mis-sense frameshift from Thr477Asn, truncates protein to 481aa |
|  |  | Two component systems | *agrC* | Accessory gene regulator C | Sensor histidine kinase | 1260 | 419 | Insertion | 768, A | Mis-sense frameshift from Asn256Lys, truncates protein to 262aa |
|  |  | Regulatory function | *stp1* | Protein phosphatase 2C domain-containing protein | Serine/threonine specific protein phosphatase | 744 | 247 | SNP | 297, G 🡪 A | Met99Ile |
|  |  | Hypothetical | SA1264 | Conserved hypothetical protein | Unknown function | 222 | 73 | SNP | 140, G 🡪 A | Gly47Glu |
|  |  |  |  |  |  |  |  |  |  |  |
| **A9754** | **A9757** | Phopsholipid synthesis | *mprF* | Lysylphosphatidylglycerol synthetase | Oxacillin resistance related protein | 2523 | 840 | SNP | 1259, T 🡪 A | Ile420Asn |
|  |  | Two component systems | *agrC* | Accessory gene regulator C | Sensor histidine kinase | 1293 | 430 | SNP | 172, C 🡪 T | Pro58Ser |
|  |  |  | *agrA* | Accessory gene regulator A | Response regulator | 717 | 238 | Deletion | 712, A | Mis-sense frameshift from Ile238Tyr, extends protein to 259aa |
|  |  |  | *yycG* | WalK-like sensor kinase, VicK | Sensor histidine kinase | 1827 | 608 | SNP | 1412, T 🡪 C | Ile471Thr |
|  |  | Protein synthesis | *rpoB* | DNA-directed RNA polymerase B-subunit | RNA polymerase | 3552 | 1183 | SNP -1  SNP -2 | 1402, A 🡪 C  1457, C 🡪 T | Lys468Gln  Ser486Leu |
|  |  | Transporters | *vyqF* | Putative antibiotic transporter | Cell-wall associated antibiotic response protein | 702 | 233 | SNP | 355, T 🡪 A | Trp119Arg |
|  |  | Cellular processes | *purA* | Adenylosuccinate synthetase | Purine biosynthesis | 1284 | 427 | SNP | 691, T 🡪 C | Tyr231His |
|  |  |  | *bioF* | 8-amino-7-oxononanoate synthase | Unknown function | 1116 | 371 | SNP | 523, A 🡪 G | Ile175Val |
|  |  | Hypothetical | SA0363 | Hypothetical protein | Putative lipoprotein | 627 | 208 | SNP | 461, T 🡪 C | Val154Ala |
|  |  |  |  |  |  |  |  |  |  |  |
| **A8796** | **A8799** | Phospholipid synthesis | *mprF* | Lysylphosphatidylglycerol synthetase | Oxacillin resistance related protein | 2523 | 840 | SNP | 1010, C 🡪 T | Ser337Leu |
|  |  | Cellular processes | *citZ* | 2-methylcitrate synthase | Kreb’s cycle, citrate synthesis | 1122 | 373 | SNP | 661, A 🡪 G | Ser221Gly |
|  |  |  |  |  |  |  |  |  |  |  |
| **A9763** | **A9764** | Phospholipid synthesis | *mprF* | Lysylphosphatidylglycerol synthetase | Oxacillin resistance related protein | 2523 | 840 | SNP | 2476, C 🡪 T | Leu826Phe |
|  |  |  | *cls2* | Cardiolipin synthetase | Phosphatidylcholine-hydrolysing phospholipase | 1495 | 494 | SNP | 156, A 🡪 C | Leu52Phe |
|  |  | Two component systems | SA1667 | Sensor histidine kinase | Two-component signal transduction system | 1113 | 370 | SNP | 161, T 🡪 A | Leu54His |
|  |  |  |  |  |  |  |  |  |  |  |
| **A9765** | **A9766** | Phospholipid synthesis | *mprF* | Lysylphosphatidylglycerol synthetase | Oxacillin resistance related protein | 2523 | 840 | SNP | 884, C 🡪 T | Ser295Leu |
|  |  | Cellular processes | SA1291 | Glycosyl transferase | Transfers sugar moieties to teichoic acids | 1143 | 380 | SNP | 716, T 🡪 C | Val239Ala |
|  |  |  | SA0103 | Conserved hypothetical protein | Integral membrane protein, drug/metabolite exporter family protein (DMT family permase) | 873 | 290 | SNP | 204, T 🡪 C | Pro68Pro |
|  |  |  |  |  |  |  |  |  |  |  |
| **A9781** | **A9784** | Protein synthesis | *rpoB* | RpoB | β-subunit of DNA-directed RNA Polymerase | 3552 | 1183 | SNP | 1430, C 🡪 A | Ala477Asp |
|  |  | Carbohydrate metabolism | SA0248 | Glycosyl transferase | Transfers sugar moieties to teichoic acids | 1722 | 573 | SNP | 1024, T 🡪 G | Cys342Gly |
|  |  | Cellular processes | *clpX* | ATP-binding subunit ClpX | Part of ATP-dependent Clp protease | 1263 | 420 | Deletion | 168, T | Mis-sense frameshift from Lys25Asn, truncates protein to 26aa |
|  |  |  | *citZ* | 2-methylcitrate synthase | Kreb’s cycle, citrate synthesis | 1122 | 373 | SNP | 664, A 🡪 G | Thr222Ala |
|  |  |  |  |  |  |  |  |  |  |  |
|  | **A9788** | Protein synthesis | *rpoB* | RpoB | β-subunit of DNA-directed RNA Polymerase | 3552 | 1183 | SNP | 1430, C 🡪 A | Ala477Asp |
|  |  | Carbohydrate metabolism | SA0248 | Glycosyl transferase | Transfers sugar moieties to teichoic acids | 1722 | 573 | SNP | 1024, T 🡪 G | Cys342Gly |
|  |  | Cellular processes | *citZ* | 2-methylcitrate synthase | Kreb’s cycle, citrate synthesis | 1122 | 373 | SNP | 664, A 🡪 G | Thr222Ala |
|  |  |  |  |  |  |  |  |  |  |  |
|  | **A9792** | Phospholipid synthesis | *mprF* | Lysylphosphatidylglycerol synthetase | Oxacillin resistance related protein | 2523 | 840 | SNP | 884, C 🡪 T | Ser295Leu |
|  |  | Protein synthesis | *rpoB* | RpoB | β-subunit of DNA-directed RNA Polymerase | 3552 | 1183 | SNP | 1430, C 🡪 A | Ala477Asp |
|  |  | Carbohydrate metabolism | SA0248 | Glycosyl transferase | Transfers sugar moieties to teichoic acids | 1722 | 573 | SNP | 1024, T 🡪 G | Cys342Gly |
|  |  | Cellular processes | *clpX* | ATP-binding subunit ClpX | Part of ATP-dependent Clp protease | 1263 | 420 | Deletion | 168, T | Mis-sense frameshift from Lys25Asn, truncates protein to 26aa |
|  |  |  | *pdhA* | Pyruvate dehydrogenase (acetyl transferring) E1 component subunit alpha | Amino acid and nucleotide metabolism | 1113 | 370 | SNP | 505, G 🡪 A | Ala169Thr |
|  |  |  | *dprA/smf* | DNA processing protein DprA/Smf-like | DNA recombination, topoisomerase, similar to Smf family | 875 | 178 | SNP | 835, T 🡪 C | Val166Ala |
|  |  |  | *citZ* | 2-methylcitrate synthase | Kreb’s cycle, citrate synthesis | 1122 | 373 | SNP | 664, A 🡪 G | Thr222Ala |
|  |  |  |  |  |  |  |  |  |  |  |
|  | **A9798** | Phospholipid synthesis | *mprF* | Lysylphosphatidylglycerol synthetase | Oxacillin resistance related protein | 2523 | 840 | SNP | 182, G 🡪 T | Gly61Val |
|  |  | Protein synthesis | *rpoB* | RpoB | β-subunit of DNA-directed RNA Polymerase | 3552 | 1183 | SNP | 1430, C 🡪 A | Ala477Asp |
|  |  | Carbohydrate metabolism | SA0248 | Glycosyl transferase | Transfers sugar moieties to teichoic acids | 1722 | 573 | SNP | 1024, T 🡪 G | Cys342Gly |
|  |  | Cellular processes | *whiA* | Conserved hypothetical protein | Contains domains homologous to sporulation factor WhiA | 945 | 314 | SNP | 535, G 🡪 T | Gly179Stop, truncates protein to 179aa |
|  |  |  | *yjbH* | Conserved hypothetical protein | Polyketide biosynthesis (dithiol-disulfide isomerase family) | 807 | 268 | Insertion | 422, A | Mis-sense frameshift from Ser141Lys, truncates protein to 141aa |
|  |  |  | *ylxS* | Conserved hypothetical protein (uncharacterised BCR, Yhbc family) | Contains YlxS-like domain from Bacillus subtilis, no known function (Yhbc family) | 468 | 155 | SNP | 446, C 🡪 A | Ala149Glu |
|  |  |  | *citZ* | 2-methylcitrate synthase | Kreb’s cycle, citrate synthesis | 1122 | 373 | SNP | 664, A 🡪 G | Thr222Ala |
|  |  |  |  |  |  |  |  |  |  |  |
| **Lab derived pairs** | |  |  |  |  |  |  |  |  |  |
| **A8115** | **A10135** | Phospholipid synthesis | *pgsA* | CDP-diacylglycerol-glycerol-3-phosphate 3-phosphatidyltransferase | Glycerophospholipid catalysis | 579 | 192 | SNP | 191, C 🡪 T | Ala64Val |
|  |  | Transporters | *norB* | Transmembrane efflux protein | Small molecule transporter | 1392 | 463 | SNP | 939, T 🡪 A | Val313Val |
|  |  |  |  |  |  |  |  |  |  |  |
| **A8115** | **A10151** | Phospholipid synthesis | *cls2* | Cardiolipin synthetase | Phosphatidylcholine-hydrolysing phospholipase | 1485 | 494 | SNP | 98, C 🡪 A | Thr33Asn |
|  |  |  |  |  |  |  |  |  |  |  |
| **A8115** | **A10152** | Phospholipid synthesis | *pgsA* | CDP-diacylglycerol-glycerol-3-phosphate 3-phosphatidyltransferase | Glycerophospholipid catalysis | 579 | 192 | SNP | 191, C 🡪 T | Ala64Val |
|  |  |  |  |  |  |  |  |  |  |  |
| **A8117** | **A10136** | Phospholipid synthesis | *pgsA* | CDP-diacylglycerol-glycerol-3-phosphate 3-phosphatidyltransferase | Glycerophospholipid catalysis | 579 | 192 | SNP | 530, C 🡪 T | Ser177Phe |
|  |  | Regulatory functions | *tcaR* | HTH-type transcriptional regulator | Associated with teicoplanin-resistance | 456 | 151 | SNP-1  SNP-2 | 206, T 🡪 G  306, A 🡪 T | Ile69Ser  Lys95Asn |
|  |  |  |  |  |  |  |  |  |  |  |
| **A8117** | **A10153** | Phospholipid synthesis | *pgsA* | CDP-diacylglycerol-glycerol-3-phosphate 3-phosphatidyltransferase | Glycerophospholipid catalysis | 579 | 192 | SNP | 191, C 🡪 T | Ala64Val |
|  |  | Amino acid metabolism | *asd* | Aspartate semialdehyde dehydrogenase | Aspartate synthesis | 990 | 329 | SNP | 280, A 🡪 G | Asn94Asp |
|  |  |  |  |  |  |  |  |  |  |  |
| **A8117** | **A10154** | Phospholipid synthesis | *pgsA* | CDP-diacylglycerol-glycerol-3-phosphate 3-phosphatidyltransferase | Glycerophospholipid catalysis | 579 | 192 | Insertion | 224, TGGGGA | Mutates Lys75 to Asn and inserts Gly76 and Glu77 in-frame |
|  |  | Cell wall function | *ebh* | Extracellular matrix binding protein | Fibronectin binding protein associated with bacterial cell wall | 31422 | 10473 | Deletion | 29452, T | Mis-sense frameshift from Lys9818Asn, truncates protein to 9822aa |
|  |  | Regulatory functions | *tcaR* | HTH-type transcriptional regulator | Associated with teicoplanin-resistance | 456 | 151 | SNP-1  SNP-2 | 206, T 🡪 G  306, A 🡪 T | Ile69Ser  Lys95Asn |
|  |  |  |  |  |  |  |  |  |  |  |
| **A5948** | **A6658** | Phospholipid synthesis | *mprF* | Lysylphosphatidylglycerol synthetase | Oxacillin resistance related protein | 2523 | 840 | SNP | 2476, C 🡪 T | Leu826Phe |
|  |  |  |  |  |  |  |  |  |  |  |
| **A5948** | **A10155** | Phospholipid synthesis | *cls2* | Cardiolipin synthetase | Phosphatidylcholine-hydrolysing phospholipase | 1485 | 494 | SNP | 98, C 🡪 A | Thr33Asn |
|  |  |  |  |  |  |  |  |  |  |  |
| **A5948** | **A10156** | Phospholipid synthesis | *pgsA* | CDP-diacylglycerol-glycerol-3-phosphate 3-phosphatidyltransferase | Glycerophospholipid catalysis | 579 | 192 | SNP | 176, T 🡪 A | Val59Asp |
|  |  | Transporters | *mnhC* | Monovalent cation/H+ antiporter subunit C | Subunit C of ionic resistance antiporter complex | 399 | 132 | SNP | 329, C 🡪 A | Ala110Glu |

***^a^*** Gene name derived from either N315 or Mu50 annotation.

***^b^*** Sizes of the gene or protein are based on the susceptible strains of *S. aureus*.

***^c^*** Nucleotide and amino acid mutations are those identified in the daptomycin-exposed strains compared to their isogenic parent strains.

***^d^*** SNP – Single nucleotide polymorphism.
