# Supplementary material for: Whole Genome Characterization of the Mechanisms of Daptomycin Resistance in Clinical and Laboratory Derived Isolates of Staphylococcus aureus
Source: PLoS One. 2012 Jan 6;7(1):e28316. doi: 10.1371/journal.pone.0028316 (PMC3253072; doi:10.1371/journal.pone.0028316)
Supplement: Text S1 — More detailed methods. (DOCX) [file pone.0028316.s003.docx]

**SUPPLEMENTARY MATERIAL**

**Materials and Methods**

**Whole genome sequencing**

For assembly annotation, GeneMark, Glimmer3, MetaGene, and Zcurveb were used to predict *ab initio* gene models. An evidence-based approach constructed open reading frames (ORFs) from BLASTX hits with the NCBI NR protein database using all BLAST hits with e-values better than 1e^-10^ as BLAST evidence. Where available, well-curated annotations from reference genomes were transferred to genome assemblies to improve automated annotation. *Ab initio* predictions, models generated using BLASTX hits against the NCBI NR protein database, transferred reference gene models, and manual gene models were clustered into potential gene loci. For each locus, the most likely non-conflicting gene models were selected based on the best available evidence. Discrepant gene models were manually reviewed.

**Membrane lipid analysis**

The membrane extract from each strain was resuspended in a mobile phase solution containing known concentrations of internal standards. The LC-MRM setup included use of an Alltech Saphira 50 mm × 1 mm internal diameter, 5-µm diol column with a mobile phase consisting of 84.8:14.8:0.5 (vol/vol/vol) (chloroform:methanol:water) + 20 mM ammonium acetate and 0.1% vol acetic acid with a flow rate of 150 µl/min with a 1 µl injection volume. MS/MS fragmentation reactions were detected by an API4000 Qtrap (Applied Biosystems) corresponding to loss of PG [M-172]^+^ of C30:0 through C36:0 PG and losses of L-PG [M-300]^+^ for C30:0 through C36:0 L-PG. Deuterated and nondeuterated standards were used to generate a calibration curve for the phospholipids being assessed. The PG and L-PG content were reported as the respective sum of the measured molecular species (C30:0 to C36:0). The phosphorus content of the extract was determined to normalize total PG and L-PG concentrations using modified methods from [1].

**References**

1. <http://www.avantilipids.com/index.php?option=com_content&view=article&id=1686&Itemid=405>
